# 

**Published:** 1879-08

**Authors:** 


### BOOKS AND PAMPHLETS RECEIVED.

Pocket Therapeutics and Dose Book. By Morse Stewart, jr., B.A., M.D. Second Edition. Revised and Enlarged. Cl., pp. 263. Detroit: G. D. Stewart, 1878.

Sixteenth Annual Report of the New York Society for the Relief of the Ruptured and Cripple. May, 1879.

Third Annual Report of the State Board of Health of the State of Wisconsin for the year ending Dec. 31, 1878. Paper; pp. 183.

The American Medical College Association. Third Annual Meeting, held at Atlanta, Ga., May 3 and 5, 1879.

Constitution and List of Members of the American Public Health Association.

On Some Points in Connection with the Treatment of Sterility. By A. Reeves Jackson, A.M., M.D. Reprint from Vol. III. Gynæcological Transactions.

Notes on Intra-Ocular Lesions Produced by Sunstroke. By F. C. Hotz, M.D.

An Argument made before The American Medical Association at Atlanta, Ga., May 7, 1879. By Edward S. Dunster, M.D. Reprint from *Physician and Surgeon*, June, 1879.

The Casual Lesions of Puerperal Convulsions. A Paper read before the Pathological Society of Philadelphia, April 28, 1878, and reprinted from the Transactions. By James Tyson, M.D. Philadelphia: J. B. Lippincott & Co.

Atlas of Histology, Part IV. By E. Klein, M.D., F.R.S., and E. N. Smith, L.R.C.P., M.R.C.S. Chicago: W. T. Keener; Philadelphia: J. B. Lippincott & Co.

The Future Influence of the Johns Hopkins Hospital on the Medical Profession of Baltimore. By John Van Bibber, M.D.

Transactions of the State Medical Society of Arkansas, at its Fourth Annual Session.

---

A PECULIARITY OF THE RICKETY PELVIS.—Prof. Depalu (*Gaz. des Hôp.*) has called attention to a deformity of the rickety pelvis, which is only occasionally observed. A pelvis thus affected presents, at certain points of the upper aperture, besides a contraction, some sharp, bony lamellæ, capable of cutting like a knife, corresponding in position to certain muscular insertions at the symphysis pubis, or the ilio-pectineal eminence. These lamellæ cause serious mischief; for on the head of the child pressing its uterine covering against their sharp edges, an incision or even true perforation of the uterine wall may be the consequence. In three such cases D. had known a true hole to have been produced, establishing a communication between the cavity of the uterus and neighboring parts. These bony projections, hidden by tendinous or muscular insertions, cannot be detected during labor.

TAPE-WORM IN A FISH.—A fishmonger in Berlin, who had lately received a considerable supply of fishes from the German Ocean, found a tape-worm of the length of two and a half meters in a carp. The worm belonged to the class *Coryophyllacus latus*, which species is occasionally met with in the intestines of the carp, pike and some birds who mostly live on fish.—*Hosp. Gaz.*

### ANNOUNCEMENTS FOR THE MONTH.

### SOCIETY MEETINGS.

Chicago Medical Society—Mondays, August 4 and 18.

West Chicago Medical Society—Mondays, August 11 and 25.

### CLINICS.

MONDAY.

Eye and Ear Infirmary—2 p. m., Ophthalmological, by Prof. Holmes; 3 p. m., Otological, by Prof. Jones.

Mercy Hospital—1:30 p. m., Surgical, by Prof. Andrews.

Rush Medical College—2 p. m., Dermatological and Venereal, by Prof. Hyde; 3 p. m., Medical, by Dr. Bridge.

Woman's Medical College—2 p. m., Dermatological, by Dr. Maynard.

TUESDAY.

Cook County Hospital—2 to 4 p. m., Medical and Surgical Clinics.

Mercy Hospital—1:30 p. m., Medical, by Prof. Hollister.

WEDNESDAY.

Chicago Medical College—1:30 p. m., Eye and Ear, by Prof. Jones.

Rush Medical College—3:30 to 4:30 p. m., Diseases of the Chest, by Dr. E. Fletcher Ingals.

THURSDAY.

Chicago Medical College—1:30 p. m., Medical, by Prof. Quine.

Rush Medical College—3 p. m., Diseases of the Nervous System, by Prof. Lyman.

Eye and Ear Infirmary—2 p. m., Ophthalmological, by Dr. Hotz.

FRIDAY.

Cook County Hospital—2 to 4 p. m., Medical and Surgical Clinics.

Mercy Hospital—1:30 p. m., Medical, by Prof. Davis.

SATURDAY.

Rush Medical College—2 p. m., Surgical, by Prof. Gunn.

Chicago Medical College—2 p. m., Surgical, by Prof. Isham; 3 p. m., Neurological, by Prof. Jewell.

Woman's Medical College—11 a. m., Ophthalmological, by Dr. Montgomery.

Daily Clinics, from 2 to 4 p. m., at the Central Free Dispensary, and at the South Side Dispensary.

